# Baseline assessment findings of the Africa Society for Blood Transfusion Step‐Wise Accreditation Programme in 10 sub‐Saharan African countries, 2016–2018

**DOI:** 10.1111/vox.13262

**Published:** 2022-03-10

**Authors:** Udhayashankar Kanagasabai, Michael Qualls, Ray W. Shiraishi, Laura Eno, Innocent Zungu, Lesley Bust, Bakary Drammeh, Dejana Selenic

**Affiliations:** ^1^ Epidemic Intelligence Service, Division of Global HIV and Tuberculosis, CGH Centers for Disease Control and Prevention Atlanta Georgia USA; ^2^ Division of Global HIV and Tuberculosis, CGH Centers for Disease Control and Prevention Atlanta Georgia USA; ^3^ Division of Global HIV and Tuberculosis Centers for Disease Control and Prevention Yaoundé Cameroon; ^4^ Division of Global HIV and Tuberculosis Centers for Disease Control and Prevention Lilongwe Malawi; ^5^ Western Province Blood Transfusion Service Cape Town South Africa

**Keywords:** accreditation, AfSBT, blood transfusion systems

## Abstract

**Background and Objectives:**

The accreditation of blood services promotes continuous quality improvement in blood and transfusion services. The Africa Society for Blood Transfusion (AfSBT) conducted 20 baseline assessments of National Blood Transfusion Services (NBTS) or blood banks as part of the Step‐Wise Accreditation Programme (SWAP) in 10 sub‐Saharan African (SSA) countries from 2016 to 2018. This paper aims to elucidate the process and findings of the baseline assessments.

**Materials and Methods:**

This is a descriptive study of 20 baseline assessments of NBTS. Eleven sections of the AfSBT assessment were reviewed, and 48 out of 68 standards and 356 out of 466 criteria were assessed. Each standard was assigned a value of 1 if it was fully achieved, 0.5 if partially achieved and 0 if not achieved. We defined average section scores >75% as having ‘met AfSBT Standards’, ≤25% as not meeting standards, 26%–50% as needs major improvement, and 51%–75% as needs some improvement and >75% as meets standards.

**Results:**

The AfSBT SWAP standards were met in 4 out of the 11 sections: donor management, blood collection, component production and compatibility testing. Three sections were determined to need some improvement (quality system; handling, transport and storage and testing of donated blood), and three sections were determined to need major improvement (haemovigilance, blood administration and national blood service accreditation). One section (receipt, ordering, and issuing of blood) did not meet standards.

**Conclusion:**

Despite improvements in the quality of blood services in SSA over the past two decades, governments may consider the importance of prioritizing investments in NBTS, ensuring these institutions meet international accreditation standards that are aligned with safe blood transfusion services.

**Highlights**

- Understanding of the AfSBT SWAP assessments provides an opportunity for NBTS in SSA to improve and achieve accreditation.
- Findings from the AfSBT SWAP assessments highlights key areas for improvement of National Blood Transfusion Services in 10 SSA countries.
- Standards such as haemovigilance, blood administration, national blood service accreditation, and the receipt, ordering of blood would benefit from much improvement.

## INTRODUCTION

Blood transfusions can be a critical and lifesaving intervention, especially in low‐resource settings where conditions requiring transfusions, such as malaria, and post‐partum haemorrhage, are often associated with high mortality and morbidity [1]. National Blood Transfusion Services (NBTS) are an integral component of resilient healthcare systems [2, 3, 4]. An efficient NBTS, that provides safe and adequate blood is a fundamental component for any healthcare delivery system and is critical for establishing safe, adequate and high‐quality blood and transfusion services [5]. In 1975, the World Health Assembly first highlighted the global need for adequate safe blood, which has led to prioritization globally and at the level of national health systems [6]. However, of the estimated 112.5 million units of blood collected in 2013, approximately 5.6 million units were collected in the World Health Organization (WHO) Africa region which makes up 16% of the global population, accounting for only about 4% of global donations [6].

In 1994, the WHO regional committee for the Regional Office for Africa (AFRO) expressed concern that, out of 44 countries in the Africa region, only 10 had the appropriate policies and systems in place to support the safety of blood transfusion in their respective countries [7]. In an effort to address these concerns, the WHO proceeded to adopt the Resolution AFR/RC44/R12 (in 2001), which encouraged member countries ‘to enact safe blood policies and mobilize resources for the development of the infrastructure of blood transfusion services in their countries’ [7]. The WHO recommends that all activities that are associated with the process of blood collection, testing, processing, storage and distribution be coordinated at the national level through an effective organization and integrated blood supply networks [8, 9].

Accreditation is defined by Hindawi as ‘a non‐governmental, voluntary process whereby an agency or association grants public recognition to an organization for having met certain established standards’ [10]. These established standards may be determined through either initial and periodic evaluations that involve submitting a self‐evaluation report, site inspection or by a team of experts conducting an evaluation by an independent board or commission [10, 11, 12]. NBTS in Africa operate at widely different levels of development from high to more basic levels. Consequently, most African countries have found international blood transfusion standards and requirements too complex and expensive to adopt in resource‐constrained settings [7].

To address the challenges in meeting accreditation standards, the Africa Society for Blood Transfusion (AfSBT), which was established in 1997, developed blood transfusion standards relevant to Africa [7]. The mission of the AfSBT is to, ‘advocate for the highest ethical and professional standards, practices and skills in blood transfusion across the African continent, enabling safe, universally accessible and sustainable national blood programs in participating countries’ [13].

The AfSBT Standards are evidence‐based best practices in blood transfusion and were initially based on the WHO Aide Memoire for Blood Safety. The Standards for the AfSBT Step‐Wise Accreditation Program (SWAP) were initially developed in 2013 by a sub‐group of the Task Team for Accreditation established by the AfSBT with guidance from the American Association of Blood Banks. The primary goal of the Standards is to provide a benchmark that is achievable for the accreditation of blood banks and NBTS to maintain and improve the quality and safety of blood transfusion infrastructure, systems, and practices in Africa [10, 13]. Since AfSBT's establishment in 2013, over 20 countries have been engaged in the process of acquiring accreditation.

The AfSBT Standards are applicable to blood transfusion services or individual health facilities that perform the following blood processing functions: mobilization, recruitment, selection and screening of blood donors; collection of blood, processing of blood into blood products, testing of blood and blood products for group and transfusion transmissible infectious disease, pre‐transfusion/compatibility testing; and the storage, handling, transportation and distribution of blood and products [13]. The AfSBT accreditation process is entirely voluntary and consists of using a set of standards which is made up of three progressively more uncompromising levels of compliance, required as follows: Step 1; meeting minimum (basic) level certification, Step 2: meeting intermediate level certification (the intermediate step includes progressively more detailed requirements and standards than basic but less than full requirements), and Step 3: full accreditation at international standard. The entire accreditation process is further comprised of a series of assessments conducted by external independent blood transfusion experts: baseline assessment, progress assessment, formal assessment, re‐assessment, surveillance assessment and repeat assessments [13]. A compliance chart defines the evidence required to achieve compliance at each of the three steps. The baseline assessment is the tool used to determine compliance with requirements of the AfSBT Standards at the initiation of the accreditation process. Prior to any external assessments, a blood transfusion service utilizes a gap analysis to perform a self‐assessment and determines the most appropriate step that best correlates with its performance concomitant with AfSBT assistance. The AfSBT supports the country in conducting an initial baseline assessment and an action plan is developed to address the deficiencies in obtaining the standards, with established timelines for rectifying said deficiencies. Once the gaps have been addressed, follow‐on assessments can be conducted so that certification at Steps 1 or 2 or full accreditation at Step 3 can be achieved. To receive full accreditation at an international standard, the blood service must comply with all the required criteria of the standards. The AfSBT accreditation is valid for 3 years.

This paper aimed to summarize and describe the findings from 20 AfSBT baseline assessments of the AfSBT and identify those sections, standards and required criteria that were hard to achieve by NBTS.

## METHODS

We conducted a descriptive study of baseline assessments conducted by AfSBT at 20 blood transfusion centres (sites) supported by the NBTS in 10 SSA countries between July 2016 and December 2018. For this study, sites are defined as those blood centres that are authorized by the NBTS to conduct blood collection, testing, processing, storage and distribution; some countries had more than one site that was assessed.

The baseline assessments were conducted by an external team comprising of blood transfusion experts employed or hired by the AfSBT. The baseline assessments consist of the first step in the SWAP process for achieving accreditation. Baseline assessments were conducted using three modalities: interviews, direct observation and record review.

The baseline assessment consists of 12 sections that cover the whole blood transfusion process from vein to vein. The sections are made up of 68 standards, which are sub‐divided into 466 individual required criteria. In this paper, section 12 of the standards (Plasma Provided for Fractionation) and its standards (14) were excluded from the analysis across all 20 assessments as this function is considered ‘elective’ and was not assessed uniformly at all sites. This section is also not considered a minimum requirement for a well‐organized and safe blood transfusion service by the WHO (Aide Memoire) [9, 11, 12].

### Data extraction

The authors used the WHO Aide Memoire which contains a checklist of key components that must be included in a well‐organized NBTS to purposively identify 356 (356/466) criteria and 48 (48/54) standards, which represented the remaining 11 (11/12) sections for analysis (Table 1) [9, 14]. The number of standards assessed per section ranged from 1 to 12. The data from these 20 baseline assessments were extracted from the baseline assessments using a standardized structured questionnaire comprising the 356 criteria in Microsoft Excel.

**TABLE 1:** African Society for Blood Transfusion Accreditation Standards — Section, required criteria, priority indicators and standards

Section (*N* = 12)		Standards (*N* = 68)	Number of standards assessed (*N* = 48)	Required criteria (*N* = 466)	Priority criteria assessed (*N* = 356)
I	Quality system^a^	12	12	177	134
II	Blood Donor Management^b^	5	5	17	17
III	Collection of Blood from Donor^c^	7	7	37	37
IV	Handling, Transport and Storage^d^	4	4	17	17
V	Testing of Donated Blood^e^	3	3	22	22
VI	Blood Component Production^f^	3	1	30	24
VII	Receipt Ordering, Selection and Issuing of Blood and Blood Components^g^	4	4	28	28
VIII	Compatibility Testing^h^	4	2	39	29
IX	Haemovigilance and Clinical Interface^i^	4	4	16	16
X	Blood Administration^j^	4	2	18	13
XI	National Blood Service Accreditation^k^	4	4	19	19
XII	Requirements if plasma is provided for fractionation^l^	14	‐	46	‐

^a^ The organization's structure, responsibilities, policies, procedures and resources established and approved by top management to achieve quality.

^b^ Entails several key processes that together aim at providing for the proper number of donations and blood product needed.

^c^ Process that includes recruitment, donor invitation, donor selection, donation procedures and donor retention.

^d^ Procedures to ensure that blood and blood components are handled, stored and transported in a manner that prevents damage and meets specific requirements.

^e^ Process for performing blood group serology and testing for infectious diseases carried out on donated specimens.

^f^ Methods that ensure the quality and safety of blood components, including aliquots and pooled components.

^g^ Procedures to check all incoming blood and blood components from another center against delivery document for number and group of components.

^h^ Testing of each blood specimen from a potential recipient for ABO group, for Rhesus factor type and for clinically significant antibodies.

i Adverse events related to blood donation process are assessed, investigated and monitored.

^j^ Procedures for administering blood and blood components.

^k^ Requirements for a well‐organized, nationally coordinated blood transfusion service to ensure availability of safe blood that is accredited.

^l^ Excluded from analysis.

### Statistical analysis

Each of the 48 standards was assigned a value of 1 if it was achieved fully, 0.5 if achieved partially and 0 if not achieved [13]. For each site, standard scores were summed by section, and the mean, median and the first and third quartile scores for each section were calculated across the 20 baseline assessments. These summary measures were then divided by the maximum score available for each section (Table 1) and expressed as percentages of the total available score. For the purpose of this paper, we determined that sites that had section scores ≤25% as not meeting standards, >26%–50% as needs major improvement, >51%–75% as needs some improvement and >75% as meets standards [15].

### Ethical review

Data analysed in this report were routinely collected for program monitoring, improvement and evaluation purposes only. This non‐research activity was reviewed by centers for disease control and prevention (CDC) and was conducted consistent with applicable federal law and CDC policy.

## RESULTS

**FIGURE 1:**
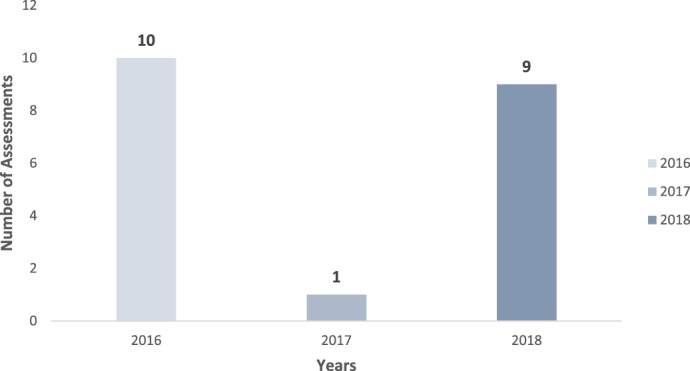
Number of AfSBT baseline assessments — 2016–2018. AfSBT, African Society for Blood Transfusion

Baseline assessments at 20 blood transfusion sites in 10 SSA countries were conducted over a period of 3 years (Figure 1). Ten baseline assessments were conducted in 2016, one in 2017 and nine in 2018. Due to funding constraints, only one baseline assessment was conducted in 2017. The 20 baseline assessments represent the NBTS of 10 PEPFAR‐supported countries that engaged the AfSBT in the accreditation process. All sites managed by the NBTS in the 10 countries were assessed as part of the accreditation process, as such, some country's NBTS had multiple transfusion sites that participated in the analysis. All sites were located in urban centres, and none had achieved prior accreditation. However, for the purpose of this paper, we analysed the scores from each site individually.

Four sections (Blood Donor Management, Collection of Blood from Donors, Blood Component Production and Compatibility Testing) out of the 11 (Table 2) had mean and median percentages exceeding 75%. These four sections had high average scores indicating that these sections were performing well and had met the standards of that section (Table 2). Averages were as follows: blood component production (97%); collection of blood from donors (90%); compatibility testing (80%) and blood donor management (78%). Two sections (Blood Component Production and Collection of Blood from Donors) performed above (75%) at 19 out of 20 sites (Table 3).

**TABLE 2:** Average score of assessed standards within sections of the AfSBT^a^ baseline assessment

Section		Score		Average percent (%)	Median percent (%)	Quartiles	
		Average	Maximum			1	3
I	Quality System^a^	8	12	66.7	69	61	72
II	Blood Donor Management^b^	3.9	5	78.0	80	68	90
III	Collection of Blood from Donor^c^	6.3	7	90.0	86	84	100
IV	Handling, Transport and Storage^d^	2.8	4	75.0	75	50	88
V	Testing of Donated Blood^e^	2	3	66.7	67	63	83
VI	Blood Component Production^f^	0.97	1	97.0	100	100	100
VII	Receipt Ordering, Selection and Issuing of Blood and Blood Components^g^	0.6	4	15.0	13	13	13
VIII	Compatibility Testing^h^	1.6	2	80.0	100	50	100
IX	Haemovigilance and Clinical Interface^i^	1.9	4	47.5	50	38	50
X	Blood Administration^j^	0.6	2	30.0	25	0	50
XI	National Blood Service Accreditation^k^	1.8	4	45.0	38	38	50
XII	Requirements if plasma is provided for fractionation^l^	‐	‐	‐	‐	‐	‐

Abbreviation: AfSBT, African Society for Blood Transfusion.

^a^ The organization's structure, responsibilities, policies, procedures and resources established and approved by top management to achieve quality.

^b^ Entails several key processes that together aim at providing for the proper number of donations and blood product needed.

^c^ Process that includes recruitment, donor invitation, donor selection, donation procedures, and donor retention.

^d^ Procedures to ensure that blood and blood components are handled, stored and transported in a manner that prevents damage and meets specific requirements.

^e^ Process for performing blood group serology and testing for infectious diseases carried out on donated specimens.

^f^ Methods that ensure the quality and safety of blood components, including aliquots and pooled components.

^g^ Procedures to check all incoming blood and blood components from another center against delivery document for number and group of components.

^h^ Testing of each blood specimen from a potential recipient for ABO group, Rhesus factor type and clinically significant antibodies.

^i^ Adverse events related to blood donation process are assessed, investigated and monitored.

^j^ Procedures for administering blood and blood components.

^k^ Requirements for a well‐organized, nationally coordinated blood transfusion service to ensure availability of safe blood that is accredited.

^l^ Excluded from analysis.

**TABLE 3:** Site achievement of standards by sections of the AfSBT baseline assessment

Section		Not meeting standard (<25%)	Needs major improvement (>26%–50%)	Needs some improvement (>51%–75%)	Meet standard (>75%)	Total	
		*N* (%)	*N* (%)	*N* (%)	*N* (%)	*N*	%
I	Quality System^a^	0 (0)	2 (10)	13 (65)	5 (25)	20	100%
II	Blood Donor Management^b^	0 (0)	3 (15)	4 (20)	13 (65)	20	100%
III	Collection of Blood from Donor^c^	0 (0)	0 (0)	1 (5)	19 (95)	20	100%
IV	Handling, Transport and Storage^d^	1 (5)	5 (25)	3 (15)	11 (55)	20	100%
V	Testing of Donated Blood^e^	1 (5)	4 (20)	9 (45)	6 (30)	20	100%
VI	Blood Component Production^f^	0 (0)	1 (5)	0 (0)	19 (95)	20	100%
VII	Receipt Ordering, Selection and Issuing of Blood and Blood Components^g^	16 (80)	4 (20)	0 (0)	0 (0)	20	100%
VIII	Compatibility Testing^h^	0 (0)	6 (30)	0 (0)	14 (70)	20	100%
IX	Haemovigilance and Clinical Interface^i^	0 (0)	16 (80)	1 (5)	3 (15)	20	100%
X	Blood Administration^j^	10 (50)	9 (45)	0 (0)	1 (5)	20	100%
XI	National Blood Service Accreditation^k^	0 (0)	17 (85)	3 (15)	0 (0)	20	100%
XII	Requirements if plasma is provided for fractionation^l^	‐	‐	‐	‐	‐	‐

Abbreviation: AfSBT, African Society for Blood Transfusion.

^a^ The organization's structure, responsibilities, policies, procedures and resources established and approved by top management to achieve quality.

^b^ Entails several key processes that together aim at providing for the proper number of donations and blood product needed.

^c^ Process that includes recruitment, donor invitation, donor selection, donation procedures, and donor retention.

^d^ Procedures to ensure that blood and blood components are handled, stored and transported in a manner that prevents damage and meets specific requirements.

^e^ Process for performing blood group serology and testing for infectious diseases carried out on donated specimens.

^f^ Methods that ensure the quality and safety of blood components, including aliquots and pooled components.

^g^ Procedures to check all incoming blood and blood components from another center against delivery document for number and group of components.

^h^ Testing of each blood specimen from a potential recipient for ABO group, Rhesus factor type and for clinically significant antibodies.

^i^ Adverse events related to blood donation process are assessed, investigated and monitored.

^j^ Procedures for administering blood and blood components.

^k^ Requirements for a well‐organized, nationally coordinated blood transfusion service to ensure availability of safe blood that is accredited.

^l^ Excluded from analysis.

Three sections (Quality Systems; Handling, Transportation, and Storage and Testing of Donated Blood) out of the 11 had mean and median percentages between >51% and 75%. These three sections had averages that indicated that these sections needed some improvement (i.e., >51%–75%; Table 2). Averages were as follows: handling, transport, and storage (75%); quality system (67.7%); and testing of donated blood (67.7%) (Table 2). One section (Quality System) performed above 51% at 18 out of 20 sites (Table 3).

Three sections (Haemovigilance and Clinical Interface, Blood Administration and National Blood Service Accreditation Requirements) out of the 11 had averages that indicated that these sections needed major improvement (i.e., >26%–50%; Table 2). Averages were as follows: haemovigilance and clinical interface (47%), blood administration (30%) and national blood service accreditation requirements (45%). Two sections (Haemovigilance and Clinical Interface and National Blood Accreditation Requirements) performed above 26% at 16 out of 20 and 17 out of 20 sites, respectively (Table 3).

One section (Receipt, Ordering, Selection, and Issuing of Blood and Blood Components) scored below 25% (considered not meeting the standards). This section performed below 25% at 16 out of 20 sites that completed the baseline assessment (Table 3).

## DISCUSSION

Across the 10 countries and 20 sites that underwent the baseline assessment, which is the first stage of the AfSBT SWAP, standards were met in 4 out of the 11 sections. This is the first report of the baseline assessment findings of the AfSBT SWAP. Currently, there is a paucity of information on accreditation and quality standards of NBTS in SSA. Prior to the AfSBT SWAP, most international standards were considered inapplicable or too difficult to implement in low‐ and middle‐income country (LMIC) settings [2, 16, 17]. Our findings emphasize the importance of accreditation standards to ensure the availability of safe and adequate blood supplies in SSA [12]. At the time of data collection and analysis, only four countries in SSA had achieved full accreditation while several others were at various stages of the SWAP process [3]. Of the 10 countries assessed, four ranked within the medium human development index (HDI) category, while the other six all ranked in the low HDI category, highlighting the importance of the AfSBT SWAP as a tool for low resource settings [4, 5].

Despite a good performance in four sections of the stepwise accreditation standards assessed by the AfSBT, essential components for providing safe blood to the public such as quality systems; testing of donated blood; receipt, ordering, and issuing of blood components; haemovigilance and NBTS accreditation, still require significant improvement.

Safe and available blood is particularly important for reducing maternal mortality, under‐five mortality associated with malaria and trauma‐associated mortality [18, 19, 20, 21]. However, the availability of safe blood for use within the healthcare system depends upon routinely meeting high‐quality standards established by AfSBT. Accreditation is considered an important approach for improving the quality of any NBTS. The AfSBT SWAP was developed to address the lack of applicable quality standards for the African context [7]. Since its establishment in 1997, and as of 2018, AfSBT has conducted 20 baseline assessments at 20 sites and in 10 countries across the continent. Regardless of the progress made in the field of blood safety in Africa by AfSBT, little has been published about the SWAP and its achievements [13, 16]. The International Organization for Standards (ISO) is the most common standard used by medical laboratories; however, this is often an expensive and a laborious process for LMIC. The more recent WHO‐AFRO Stepwise Laboratory Quality Improvement Process Towards Accreditation, like the AfSBT SWAP, has been an attempt to make the process more achievable and affordable [22]. Findings from evaluations of laboratory programs highlight the importance of accreditation and its contribution towards a resilient healthcare system and quality improvement [23, 24]. The findings from this report are important for countries to learn from and to encourage the widespread use of the AfSBT SWAP standards across the respective NBTS on the African continent. It is important to highlight that the analysis conducted focuses on select priority standards and does reflect an in‐depth review of all standards assessed by the AfSBT SWAP [9, 16].

The primary goal of an NBTS is to ascertain that there is a national supply of safe and appropriate blood products for the population in need at the right time and place [25, 26]. To ensure safe blood requires the NBTS to have a well‐delineated system that prioritizes quality from vein to vein. To safeguard this process, a quality system which identifies a set of standards that the organization commits to achieving, is required [27]. There are two key elements to any quality system, which are technical standards (which define what needs to be achieved) and quality standards (that help determine how technical standards are met) [27, 28]. Technical standards (what needs to be achieved, e.g., NBTS organogram) are easier to achieve with multiple international and regional guidelines available, however, quality standards (e.g., minimum equipment requirements) require much more rigorous support and investments [27, 29, 30]. Among the AfSBT SWAP Standards, the quality system section consists of 12/68 standards and 177/466 required criteria, respectively. For this study, we assessed 134/177 required criteria within the quality section, based on WHO recommendations [9]. The quality standards help ensure that the NBTS provides safe and infection‐free blood per international standards. Notably, the average baseline score for the quality system section of the AfSBT SWAP standards was only 66.7%, suggesting considerable room for the continuous quality improvement and need for countries to prioritize and invest in strengthening NBTS quality systems.

Behavioural screening of blood donors and pre‐transfusion testing are some of the most important tasks undertaken by an NBTS. Behavioural screening includes the recruitment of low‐risk donors and repeat donors, while screening out high‐risk donors who are subsequently deferred [31, 32]. In 2016, the percentage of blood donations that were screened and found to be HIV positive in seven sub‐Saharan African countries remained higher than the WHO target of <1% [3, 33, 34]. The WHO Global Database for Blood Safety (GDBS) 2016 report stated that in high‐income countries, 99.6% of the donations were screened following basic quality‐assured procedures, compared to 97% in upper middle‐income countries, 81% in lower middle‐income countries and only 66% in low‐income countries [6]. The overall average score of 66.7% for the testing section of the AfSBT SWAP Standards supports GDBS findings underscoring the need to improve NBTS testing practices. These findings are significant given that prevalence of blood‐borne diseases such as HIV, hepatitis B and C are highest in LMICs [34, 35, 36]. If infectious disease such as HIV, hepatitis, tuberculosis and malaria transmission in healthcare settings are to be successfully prevented, increased investments are needed to improve testing processes in NBTS and laboratories. The testing process can be broken down into three phases: the preanalytical, analytical and postanalytical phases [27]. Improvement in all three of these phases requires investments in training, equipment, systems, internal proficiency testing and appropriate external quality assurance schemes [37]. The Maputo Declaration calls for collaboration and coordination between host governments, donors and partners to ensure resilient and sustainable laboratory systems in LMICs, and such initiatives are also applicable to transfusion systems [38].

Within a transfusion system, the proper management of blood products and inventory of blood ensure that products are available as needed, and are suitable for blood transfusions for those patients who need it most while avoiding wastage of a valuable resource. Traceability from blood donor to recipient is of utmost importance for blood components. Findings from this assessment show that one of the most important aspects of the NBTS to strengthen is that of the receipt, ordering, selection, and issuing of blood and blood components (15%). The availability of well‐structured systems and processes for the management and control of blood products produced and distributed by a transfusion centre is critical. Several studies and policy documents emphasize the need for inventory management systems that can track blood from the donor to that of the recipient [12, 27, 37, 39, 40]. Haemovigilance aims to detect adverse events associated with blood products, both with donors and recipients [17, 25, 41]. The findings from the baseline assessment further emphasize the lack of adequate haemovigilance and clinical interface (47.5%) and blood administration (30%). A similar finding was reported in 2013, which stated that ‘only 13 out of 46 African countries had a national hemovigilance system’ [36]. A successful and effective haemovigilance system requires reporting of set indicators, implementation, and the regular monitoring and evaluation of these systems [25, 42]. However, for these systems to be effective, the literature and best practices suggest the use of electronic blood safety systems that are connected to the hospitals and health centres that request and use blood [43].

This evaluation had several limitations. First, due to the length and complexity of the AfSBT SWAP Standards, we were not able to compare each individual criteria across the 20 different assessments. Second, these baseline assessments were undertaken at different points in time, at different sites and in different countries across SSA and, therefore, findings cannot be generalized to all countries. Third, the findings are from the initial baseline assessments conducted between 2016 and 2018. Since then, follow‐up assessments have been conducted and several countries have progressed to achieve Step 1 or 2 certification or full accreditation at Step 3 [13]. More countries have joined the AfSBT accreditation process since the engagement of the 10 initial countries. As such, we cannot conclusively use these findings to represent transfusion services across the African continent. Finally, all assessments were not conducted by the same assessment team as such it is possible that there might have been some degree of assessor bias that could have resulted in some baseline assessments receiving higher evaluations versus others.

In conclusion, while the AfSBT SWAP provides countries with low resources a pathway and opportunity to accomplish international accreditation much needs to be done at the NBTS to achieve all the requirements for standards and accreditation. This system acknowledges the variability of SSA blood services, healthcare systems and resources, making allowance for different levels to be considered for certification and accreditation. Organizations such as WHO, PEPFAR and the Global Fund to Fight AIDS, Tuberculosis and Malaria have provided support and technical assistance to SSA countries in an effort to improve transfusion services. However, consideration for further investments may be instrumental to improving key areas such as quality systems, testing, inventory management and haemovigilance to ensure the provision of safe and adequate blood transfusion services. Accreditation of systems provides crucial evidence to users or purchasers of a service that appropriate standards are in place. The need for all NBTS in SSA to meet international standards is one which has been emphasized by WHO since the 1990s. However, the findings from the baseline assessments show that few blood banks, or NBTS, initially met the basic requirements necessary to move to the next step of the accreditation process without first making significant improvements and investments.

## CONFLICT OF INTEREST

The findings and conclusions in this report are those of the authors and do not necessarily represent the official position of the funding agencies. The authors declare no conflict of interests.
